# 3D-Printed Polylactide-Based Implants: Influence of Processing, Radiation Sterilization and In Vivo Bioresorption on Structural and Physicochemical Material Characteristics

**DOI:** 10.3390/polym18091034

**Published:** 2026-04-24

**Authors:** Monika Dobrzyńska-Mizera, Monika Knitter, Małgorzata Muzalewska, Marek Wyleżoł, Jacek Andrzejewski, Patryk Mietliński, Bartosz Gapiński, Maciej Stagraczyński, Michał Mikulski, Alessandra Longo, Giovanni Dal Poggetto, Maria Cristina Del Barone, Maria Laura Di Lorenzo

**Affiliations:** 1 Institute of Materials Technology, Division of Plastic Processing, Poznan University of Technology, Piotrowo 3, 61-138 Poznan, Poland; monika.knitter@put.poznan.pl (M.K.); jacek.andrzejewski@put.poznan.pl (J.A.); 2 Department of Fundamentals of Machinery Design, Faculty of Mechanical Engineering, Silesian University of Technology, Konarskiego 18A, 44-100 Gliwice, Poland; malgorzata.muzalewska@polsl.pl (M.M.);; 3 Institute of Mechanical Technology, Division of Metrology and Measurement Systems, Poznan University of Technology, Piotrowo 3, 61-138 Poznan, Polandbartosz.gapinski@put.poznan.pl (B.G.); 4 4th Military Clinical Hospital with Polyclinic, Rudolfa Weigla 5, 53-114 Wrocław, Poland; 5 Artdent Dental Office, Piekarska 11-13, 62-800 Kalisz, Poland; michal.k.mikulski@gmail.com; 6 National Research Council (CNR), Institute of Polymers, Composites and Biomaterials (IPCB), Via Campi Flegrei, 34, 80078 Pozzuoli, NA, Italygiovanni.dalpoggetto@cnr.it (G.D.P.); marialaura.dilorenzo@cnr.it (M.L.D.L.)

**Keywords:** bone regeneration, bioresorption kinetics, FDM printing, personalized implants, poly(lactic acid) copolymer

## Abstract

The manuscript details the influence of high-temperature and high-shear processing, as well as radiation sterilization, on properties of bioresorbable and osteoconductive, patient-tailored alloplastic scaffolds for guided bone regeneration. Functionalized poly(l-lactide-*co*-d,l-lactide) copolymer filled with hydroxyapatite was used to produce two personalized implants for upper and lower jaw reconstruction via 3D printing. Morphology analysis (SEM, µCT), gel permeation chromatography, and thermal analysis quantified the effects of melt processing and sterilization on chain structure. Physical properties of sterilized parts, such as hardness and density, proved suitable for bone implants. Removal of the upper jaw implant after 4 months and of the lower jaw substitute after 18 months enabled monitoring of bioresorption and tissue regrowth over time. Gradual overgrowth of the implants with human tissue, initiated by the osteoconductive filler, was observed, along with time-dependent polylactide degradation, showing up to 92% molar mass reduction. The medical procedures confirmed safety, nontoxicity, non-allergenicity, and, most importantly, the tissue-forming properties of the polylactide-based formulation.

## 1. Introduction

Bones, the most abundant hard tissue in the human body, are frequently affected by diseases and fractures. This has driven the growth of the bone graft substitute market—valued at nearly USD 2.5 billion in 2024 worldwide and projected to reach almost USD 4 billion by 2033 [1]. A variety of bone graft substitutes have been developed in orthopedic practice: autografts (using the patient’s own bone), allografts (donor bone), xenografts (bone from other species), and alloplasts (man-made biocompatible materials). The latter are lab-made implantation devices that are designed and tailored for the specific needs of each patient [2], able to overcome the intrinsic limitations of other solutions, like the need for additional surgery (autografts), or the risk of rejection or transmission of pathogens (allografts and xenografts).

The alloplastic scaffolds made of bioresorbable materials serve as temporary structures to be infiltrated by native tissue, i.e., tissue regeneration proceeds simultaneously with gradual elimination of the polymer from the body [3,4]. An additional advantage of these materials over autografts, allografts, and xenografts lies in their potential for shape-customization to meet individual patient requirements. Manufacturing patient-tailored scaffolds requires the implementation of comprehensive, multi-phase advanced production processes [2]. In the case of thermoplastic alloplasts, the manufacturing processes typically involve melt blending of the polymer matrix and the filler, often via twin screw extrusion, followed by filament fabrication through single screw extrusion, implant shaping via 3D printing technique, and sterilization [2]. This implies that their production may involve high-shear forces and elevated temperatures, which may impact the structural and physicochemical properties of biomaterials, with material degradation that may be further worsened by sterilization, needed to respect standards of microbiological safety [5,6,7,8]. Although a number of studies have investigated the effects of radiation-based sterilization (e.g., gamma and electron beam) on poly(lactic acid) and its composites [9,10,11,12,13,14,15], the available data remain fragmented and often material-specific. A comprehensive understanding of how sterilization affects the performance of additively manufactured PLA-based scaffolds is still missing. This is due to the complex interplay between the processing-induced microstructure (e.g., porosity, interlayer bonding, crystallinity) and radiation-induced phenomena such as chain scission, molecular weight reduction, and post-irradiation recrystallization. Consequently, there is a clear need to further elucidate and optimize the processing–structure–property relationships in alloplastic materials, with special emphasis on the role of sterilization. In this work, we aim to address this gap by systematically investigating the combined effects of processing parameters and sterilization on the structural, thermal, and physicochemical properties of additively manufactured scaffolds.

Experimental data are gained for a 3D-printed implant made with a copolymer of poly(l-lactide-*co*-d,l-lactide) suitable for direct contact with human tissues, functionalized with a bioactive filler, hydroxyapatite (HAp), to aid osteoconductivity [16]. Incorporation of solid particles into a polymeric matrix typically requires melt-mixing techniques, such as extrusion, carried out at high temperatures, above the melting point of the polymer, which adds to the subsequent processing steps required to produce a ready-to-use medical device, mentioned above. This leads to material degradation, often observed in poly(lactide)-based thermoplastics [17,18]. Consequently, assessing the impact of processing steps on the physicochemical and functional properties of the scaffold is essential to confirm its applicability in further in vivo applications.

Upon implantation, additional changes in biocomposite structure are expected, as the polymeric matrix is progressively bioresorbed, and new tissues grow, facilitated by the osteoconductive filler. The degradation of polylactide-based (PLA) composites proceeds via a coupled mechanism involving hydrolytic polymer degradation and bioactive ceramic remodeling. Initially, hydrolysis of ester bonds in PLA leads to molecular weight reduction, local acidification, and progressive matrix erosion [19,20]. This results in the exposure of hydroxyapatite particles, which subsequently undergo partial dissolution and release Ca^2+^ and PO_4_^3−^ ions [21,22]. The local supersaturation of these ions induces the formation of calcium phosphate phases through a multistep, metastable pathway. In particular, the precipitation process typically proceeds via transient, thermodynamically unstable phases such as amorphous calcium phosphate (ACP) and octacalcium phosphate (OCP), which gradually transform into more stable crystalline phases, ultimately forming biomimetic hydroxyapatite [23,24,25,26,27]. The literature details numerous in vitro and in vivo animal studies concerning the mechanisms and kinetics of bioresorption of PLA-based materials [28,29,30,31,32,33,34,35]. However, translating these data into bioresorption kinetics in the human body remains challenging, as the process is influenced by multiple factors. The most notable ones include implant morphology, geometry, composition, and the physiological environment of the human body, which differs substantially from that recreated in vitro, or observed in animal models [2]. Currently, only limited clinical data are available regarding the kinetics of bioresorption and tissue regrowth in humans [2]. Therefore, validation of laboratory findings through clinical trials remains necessary. Such data are provided in this manuscript, thanks to the need to remove two implants before complete bioresorption.

The two implants discussed here were custom-designed to rebuild contours of upper and lower jaws defects. Their implantation procedures were successful: the implants were found to be non-toxic, biocompatible, and did not elicit local or systemic inflammatory responses. Unfortunately, during treatment, one of the implants was exposed and had to be removed 4 months after implantation. The second implant was intended to restore the missing volume of the mandibular bone to allow placement of titanium dental implants for prosthetic rehabilitation. The appropriate timing for dental implant placement after bone grafting depends on the type of graft material used, the extent of the defect, and individual healing capacity. No literature data were found for this specific case of alloplastic substitute of mandibular bone; hence, a post-implantation interval of 18 months was chosen in accordance with literature data on the expected bioresorption timeframe of PLA-based scaffolds [36]. The partially remodeled implant was exposed after 18 months for titanium dental implant insertion, but when the surgeon attempted to insert titanium implants, the bone graft fractured due to its brittleness and had to be removed. This created an opportunity to thoroughly examine the explanted material in terms of structural and physicochemical changes that occurred in the polymer undergoing resorption and remodeling over different time periods, namely 4 and 18 months for the two implants detailed here.

The experimental results and data discussion provided in this manuscript contribute to filling the literature knowledge on the kinetics of bioresorption and regrowth of implanted scaffolds in humans. Of relevance are the micro-computed tomography (µCT) findings, as µCT represents a powerful tool for monitoring changes in the internal architecture, porosity, and surface topography of 3D-printed implants subjected to in vivo bioresorption [37,38,39,40,41]. Assessment of the effect of material processing on physicochemical changes in the composite material used represents another key issue of this manuscript, especially on the effect of radiation sterilization, where limited literature data are available [8]. The goal is to tailor production processes of implants that meet patients’ needs, which may further support prediction and optimization of the outcomes of such complex devices, a key issue needed to shape future therapeutic strategies.

## 2. Experimental Part

### 2.1. Materials

The implants were produced using a commercially available copolymer of poly(l-lactide-*co*-d,l-lactide) in a ratio of 80/20 (Purasorb PLDL 8058, Corbion, Amsterdam, Netherlands), further abbreviated PLA, and 10 wt.% of synthetic hydroxyapatite particles with a chemical formula of [Ca_5_(OH)(PO_4_)_3_]_x_, abbreviated HAP, in the form of whitish powder with a defined elemental composition (CaO 54.22, P2O5 33.05, and MgO 0.11%m/m), both provided by Syntplant company (Poznan, Poland). All materials and procedures used in the study possess FDA certificate or Bioethics Committee of the Greater Poland Medical Chamber approval.

### 2.2. Preparation of the Implants

**Figure 1:**
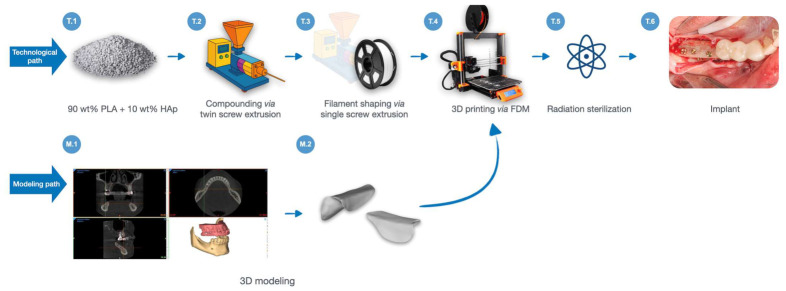
Production process of a personalized bone reconstruction implant.

Production of a patient-personalized, resorbable and osteoconductive bone reconstruction implant is a complex and multistep procedure, sketched in Figure 1 that illustrates the various tasks, sequentially numbered as T.i, and modeling (M.i) steps. Implant production starts with drying the materials under vacuum (50 °C for 24 h), and mixing in a rotary mixer Retsch GM 200, Haan, Germany (3 min at 2000 rpm) (T.1). Then, compounding in a molten state of the polymeric matrix and the appropriate amount of the filler follows via a Zamak corotating twin screw extruder operated at 210 °C and 60 rpm, to ensure appropriate distribution of the filler within the matrix (T.2). The air-cooled extrudate is pelletized and transferred for further shaping of a filament with the diameter of 1.75 mm using a Metalchem type W25-30D single screw extruder (Gliwice, Poland) equipped with a 25 mm screw and L/D = 34 (T.3) operating at 215 °C and 15 rpm. Once the filament is ready, the personalized implant is produced *via* fused deposition modeling (FDM) (T.4).

Implant production requires proper modeling to precisely fit the patient’s structural defect. Based on high-quality computed tomography (CT) imaging, a model of the diseased bone is created in the form of DICOM files (M.1). Once developed, it is further used to build the implant model via voxel haptic system (Geomagic Freeform Plus 2021, interfaced with Touch X v. 2021 model arm) (M.2). The geometric form of the implant, as well as its method of fixation, is consulted with the physician, based on specific patient’s needs, then the model is exported as .stl file for production via FDM (T.4).

The implant was 3D-printed using a desktop-type device model MK3S from (PrusaResearch, Prague, Czech Republic). The machine was equipped with a direct drive extruder and a 0.4 mm diameter brass nozzle. The printing procedure was conducted with a layer height of 0.4 mm, the nozzle temperature was set at 240 °C, while the bed platform temperature was 60 °C. The machine code (g-code) was prepared using PrusaSlicer software (2.4 edition), supplied with the printing device. During printing, a solid infill (100%) pattern was used, while the outer contour of the part consisted of two shell layers. The printing speed for the outer contour was set at 20 mm/min, while for the infill area it was 40 mm/min.

After completion of printing, the implant must be cleaned of supporting structures and sterilized. Herein, the procedure of radiation sterilization using high-energy electrons was chosen (T.5), because of its high efficiency [42]. The “Elektronika” accelerator located at the Institute of Nuclear Chemistry and Technology—Radiation Sterilization Station for Medical Devices and Allografts in Warsaw, Poland, which generates a 10 MeV electron beam with an average power of 10 kW, was used for this purpose in compliance with ISO 13485:2016 [43]. An ionizing radiation dose of 36 kGy was used.

The manufacturing and sterilization processes are expected to influence structural and physicochemical properties of the material; therefore, the composite was tested at various preparation steps: PLA-10HAp (T.2), a non-sterilized implant (T.4) and an implant subjected to radiation sterilization (T.5). The next research series concerns implants that had undergone resorption in the human body for varying periods of time, namely 4 and 18 months, to verify the impact of further in vivo progressive hydrolytic degradation on the structural and physicochemical properties of the material (T.6). The sample names and descriptions are summarized in Table 1.

### 2.3. Medical Procedures

In this paper, two medical cases are discussed to monitor the permanence of bioresorbable and osteoconductive synthetic implants in the human body for various times. The shape-personalized implants were designed to reconstruct the cleft palate (medical case no. 1) and the mandibular bone (medical case no. 2) aiming to reestablish the original anatomical bone contour and volume for further medical treatment being dental reconstruction. All experiments were conducted in accordance with the principles of the Declaration of Helsinki and approved by the Bioethics Committee at the Greater Poland Medical Chamber (approval nos. 93/2022 and 318/2022), with informed consent obtained from all participating subjects.

**Figure 2:**
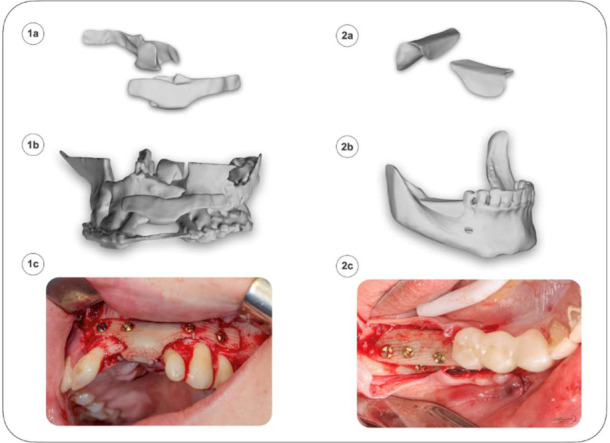
Medical cases: Patient 1—evacuation of the implant after 4 months (**1a**–**1c**); Patient 2—evacuation of the implant after 18 months (**2a**–**2c**).

*Patient 1*, a 16-year-old with a primary skeletal deformity resulting from a bilateral cleft palate, qualified for surgical reconstruction of the anterior segment of the maxillary alveolar process. As classified by Kernahan and Stark, the patient presented with a complete cleft of both the primary and secondary palate, involving the lip, alveolar ridge, as well as the hard and soft palate. Due to the exclusion of further autogenous bone augmentation as a viable option, the use of a personalized alloplastic implant was recommended as the preferred reconstructive approach (Figure 2(1a,1b). During the surgical procedure performed under local anesthesia, a full-thickness flap was elevated, and the implant was secured with four titanium screws (ϕ = 1.5 mm, ChM sp. z o.o., Juchnowiec Kościelny, Poland) and sutured in layers using 3.0 sutures (Figure 2(1c)). The immediate postoperative course was uneventful, with the patient reporting no pain, as detailed in [44]. However, in four months postoperatively, implant exposure was identified. Despite the extended duration of exposure, no clinical signs of infection or progressive enlargement of the affected area were observed. Ultimately, the detection of implant mobility necessitated surgical removal by the clinical team. The retrieved implant was subjected to further material analysis.

A 47-year-old patient (*Patient 2*) qualified for horizontal and vertical alveolar ridge augmentation in the posterior mandibular region (teeth 46–47) using a patient-specific implant designed to restore the bone volume lost over time because of tooth extractions (46 and 47) performed during childhood. During the procedure performed with local anesthesia, a full-thickness flap was elevated, and the implant was secured in place using four titanium fixation screws (ϕ = 1.5 mm, ChM sp. z o.o.). Following verification of proper flap passivity, the surgical site was meticulously closed in layers with tension-free nylon 6.0 sutures (Atramat^®^, Mexico City, Mexico) to ensure a tight seal. The postoperative course was uneventful, and the severity of symptoms in the operated area was classified as mild to moderate based on the Visual Analog Scale (VAS). Eighteen months postoperatively, the next stage of treatment—placement of dental implants—was initiated. However, during removal of the fixation screws, the implant demonstrated significant brittleness and loss of stability, rendering it unsuitable for titanium implant insertion. Consequently, a decision was made to remove the implant and proceed with bone regeneration using the technique developed by Prof. F. Khoury [45]. The retrieved implant fragments were subsequently subjected to further material characterization to assess the degree of resorption and remodeling into natural tissue.

## 3. Methods

### 3.1. X-Ray Micro-Computed Tomography (µCT)

Micro-computed tomography, as a non-destructive diagnostic technique employing X-ray radiation, enabled detailed examination of the surface topography of the samples, as well as analysis of their internal structures. The samples were mounted on a rotary stage and rotated by a constant, predefined angular step until a full 360° rotation was completed. At each angular position, the sample was exposed to X-ray radiation for a predefined exposure time of 700 ms. As a result, a set of X-ray images of the objects was obtained from different perspectives, corresponding to distinct angular positions, with the absorption of a portion of the emitted radiation by the objects producing images on a grayscale. This enabled further analysis aimed at detecting porosity, identifying material discontinuities, and characterizing the geometry of the object. The obtained images were also reconstructed via Volume Graphics STUDIO MAX 2024.2 that exploits mathematic analysis to generate three-dimensional models, which were subsequently interpreted to obtain topographic and structural data. The measurements were performed using a Phoenix V|tome|x M computed tomograph (Waygate Technologies, Hurth, Germany) with the following parameters: voltage of 120 kV, current of 100 μA, voxel size of 5 μm, and 3500 images per sample.

### 3.2. Differential Scanning Calorimetry (DSC)

Thermal properties of the PLA-based composites were investigated via differential scanning calorimetry (Phoenix 204 F1 apparatus produced by Netzsch, Selb, Germany). Approximately 4 ± 0.5 mg samples were secured in aluminum crucibles and heated from 25 °C to 200 °C at a rate of 10 K min^−1^. The main parameters that were analyzed were glass transition temperature (T_g_), cold crystallization temperature (T_cc_), melting temperature (T_m_), cold crystallization enthalpy (ΔH_Tcc_), and melting enthalpy (ΔH_Tm_).

### 3.3. Thermogravimetric Analysis (TGA)

Thermogravimetric analysis was employed to investigate the degradation profiles of the PLA-based compositions. A Netzsch TG 209 F1 apparatus (Netzsch, Selb, Germany) was set at a temperature range between 30 °C and 800 °C at a heating rate of 10 K min^−1^. An inert nitrogen atmosphere was maintained throughout all the analysis of ~8 ± 1 mg samples, placed in ceramic pans. The degradation onset temperature (T_d’onset_) was assessed at the intersection of two branch tangents of the thermogravimetric curve. The final recorded mass (m_R_) following thermal treatment at 800 °C was used to determine the residual material remaining in the sample pan. To account for instrumental drift, each thermogram was corrected by subtracting the baseline obtained from a prior run with an empty pan.

### 3.4. Gel Permeation Chromatography (GPC)

Gel permeation chromatography analyses were performed using a Malvern Panalytical (Malvern, UK) OMNISEC system, comprising the OMNISEC Resolve module (pump system, autosampler, and columns) and the OMNISEC Reveal module (multi-detector unit equipped with refractive index [RI], right-angle laser light scattering [RALS], low-angle laser light scattering [LALS], and intrinsic viscosity [IV] detectors). Samples were dissolved in chloroform containing 1% ethanol as stabilizer (Romil, Cambridge, UK), filtered on PTFE 0.22 μm, and injected at 100 µL with a concentration of ~5 mg mL^−1^. Elution was carried out at a flow rate of 0.8 mL min^−1^ through a Phenogel (Phenomenex, Torrance, CA, USA) column set, consisting of a precolumn and two analytical columns with exclusion limits of 10^6^ and 10^3^ Da. The temperature of columns and detectors was set at 35 °C. All samples were performed in duplicate, each in double injection. Molecular weight parameters were determined by triple-point calibration using polystyrene standards (Mn = 101,252 Da; Mw = 104,959 Da). Data analysis was performed with OMNISEC v11.42 software (version 105332), which provided molecular weight values (Mn and Mw) and the refractive index increment (dn/dc) of pure poly(l-lactide-*co*-d,l-lactide). Since dn/dc is specific to each polymer–solvent system [46], it was experimentally determined as 0.0252 mL g^−1^. This value was subsequently applied to calculate the effective polymer concentration injected, following removal of the insoluble fraction and expressed as recovery (%).

### 3.5. Density

To determine density, the hydrostatic method was applied in compliance with the PN-EN ISO 1183–1 standard [47]. Initial weighing of the sample was conducted in air, after which the sample was submerged in ethyl alcohol for the subsequent hydrostatic weighing. The density values were derived from Equation (1):

(1) $$\rho =\frac{m_{air}}{m_{air}-m_{liq}}\times \rho_{liq}$$

where *ρ*—sample density [g/cm^3^], *m_air_*—sample mass in the air [g], *m_liq_*—sample mass in an immersion liquid [g], and *ρ*_liq_—immersion liquid density [g/cm^3^]. A temperature of 23 °C was maintained during the measurements, which were performed using a high-precision analytical balance (resolution of 0.0001 g) equipped with a hydrostatic weighing accessory. Based on the *ρ* values, porosity (P) calculations proceeded using the following relation (Equation (2)):

(2) $$P=\left(1-\;\frac{\rho }{\rho_{th}}\right)\times 100\%$$

where *ρ*—sample density [g/cm^3^] and *ρ_th_*—density of the solid PLA-based composite material manufactured *via* compression molding technique. Conversely, compactness (C) was calculated as per Equation (3):

(3) $$C=\left(\;\frac{\rho }{\rho_{th}}\right)\times 100\%$$

### 3.6. Shore Hardness

The hardness was evaluated using the Shore D durometer method, in accordance with the ISO 868 [48]. Measurements were carried out at room temperature (23 ± 1 °C) using a calibrated digital Shore D hardness tester, produced by Sauter, Baden-Württemberg, Germany, model HBD 100-0, equipped with a Sauter test stand, model TI-D. Each sample was placed on a flat, rigid surface, and the indenter was applied perpendicularly with firm, even pressure. The reading was taken after 15 s of contact time, as specified in the standard. To ensure reproducibility and minimize local deviations, hardness was measured at five different points on each sample surface, and the results were reported as the average value.

### 3.7. Scanning Electron Microscopy

Surfaces and cross-sections of PLA-based composites were analyzed using a Quanta 200 FEG, 338 FEI scanning electron microscope (Eindhoven, The Netherlands). SEM microphotographs were collected at room temperature and a voltage of 30 kV. Before analysis, surfaces of the samples were sputter-coated with an 18 ± 0.2 nm layer of Au-Pd alloy by a MED 020 sputtering device, Bal-Tec AG, Pfäffikon, Switzerland.

## 4. Results and Discussion

### 4.1. Manufacturing and Radiation Sterilization

**Figure 3:**
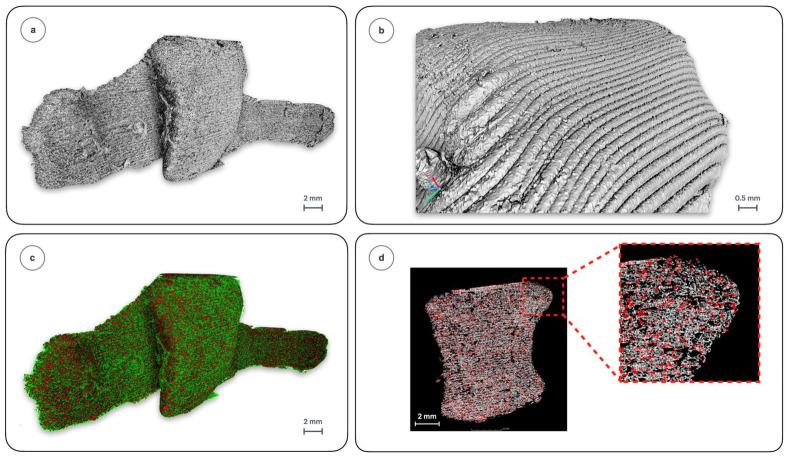
X-ray micro-computed tomography images of the *S-Ref* sample: (**a**) reference implant geometry, (**b**) surface topography, (**c**) implant geometry with visible matrix (green) and filler (red) distribution, (**d**) implant intersection together with the filler distribution (red).

X-ray micro-computed tomography (µCT) was conducted to detail the internal structure of the implant together with its surface topography, both ruled by the FDM manufacturing process. Surface parameter analysis was performed in accordance with the ISO 25178 standard [49], and summarized in Table 2 and Figure 3, where the grayscale reference model (Figure 3a), the surface topography (Figure 3b), the color-coded implant (Figure 3c) and implant intersection (Figure 3d) are presented. Tomography analysis began with height-related parameters to quantify vertical surface irregularities typical of the 3D printing process [50].

The *S-Ref* sample exhibited distinct surface irregularities and increased roughness, as evidenced by the high values of the arithmetical mean height (Sa) and root mean square height (Sq) parameters (27.33 and 35.43 µm, respectively). The statistical height parameters of the surface profile, namely the skewness (Ssk = 3.4) and kurtosis (Sku = 32.56), proved domination of sharp and irregular peaks with sudden height changes throughout the valley-like texture, attributed to the layer-by-layer deposition of the thermoplastic material. The extreme surface height parameters—maximum peak height (Sp = 103.40 µm), maximum pit depth (Sv = 249.90 µm), and maximum height of the surface (Sz = 353.40 µm)—demonstrated substantial variations in elevation between peaks and valleys, which is indicative of the layered morphology of the FDM-printed samples. Such values are rarely observed in subtractive manufacturing technologies; however, they are typical for the additive technology [51,52,53,54] and serve here to monitor the variation in surface topography caused by prolonged contact with the human body, as detailed below. The exceptionally low surface bearing index Smr (0.0011%) indicates that contact with other surfaces occurs almost exclusively at the peaks, significantly limiting the actual contact area. Complementing this surface characterization are parameters like the inverse material ratio at a defined depth (Smc = 36.85 µm) and depth at 80% material ratio (Sdc = 83.18 µm), which represent contact depth and material distribution within the profile, and further confirm the dominance of pronounced peaks and steep edges. The high values of the root mean square slope (Sdq = 1.98) and developed surface area ratio (Sdr = 39.77%) indicate a significantly increased actual surface area, clearly associated with the presence of microcracks, surface irregularities, and rounded transitions between material paths characteristic of the FDM process. The surface generated using this method exhibits a multitude of microscopic features with pronounced inclination angles, as evidenced by the previously discussed values. The autocorrelation length Sal (0.047 mm) suggests a regular distribution of surface features, which may correspond to the spacing between filament paths. The normalized surface area and roughness parameters (Shrn = 0.43, Sdrn = 0.39) further confirm that the surface exhibits a complex microstructural geometry, which sizably changes upon implantation, as quantified below.

Moreover, the grayscale used in micro-computed tomography images enables to differentiate the polymeric matrix from the HAp particles basing on the variations in their densities visible in µCT images, as marked in red color in Figure 3c,d in the sample cross-section. The analysis, performed using Volume Graphics STUDIO MAX 2024.2 software, enabled the quantification of the filler content within the sample. The filler concentration was determined to be ~10 wt.%, in accordance with the specifications established during the implant fabrication process. Furthermore, the filler is homogeneously dispersed throughout the polylactide matrix, a factor critical to the material performance and application efficacy.

It is important to note that FDM technology, besides its characteristic surface topography, also produces a porous internal structure due to the layered deposition process and the spatial arrangement of filament paths, microcracks and voids formed within the part, especially at the interfaces between successive layers. Even with full infill, the internal structure contains air pockets, resulting in reduced density and higher permeability which improves infiltration upon implantation. According to the µCT analysis of non-sterilized and sterilized implants, radiation sterilization does not cause further changes in the surface topography nor internal structure.

Sample degradation was monitored by measuring the molecular weights (M_w_ and M_n_) of non-sterilized and sterilized 3D-printed samples, in comparison with the reference PLA-10HAp sample, via gel permeation chromatography, with the results presented in Table 3. A major reduction in molar mass by 28%, in contrast to the PLA-10HAp sample, was observed for the *NS-Ref*. The PLA-10HAp was collected after melt mixing of PLA and the filler *via* twin screw extrusion (T.2, Figure 1). Further processing includes single screw extrusion (to shape the filament) and 3D printing *via* FDM (T.3, T.4, Figure 1) to obtain a personalized-shaped implant. Both processes involve elevated temperatures and shear, which are known to cause degradation in polylactides, resulting in a decrease in molar mass [55,56]. Degradation of PLA progresses via chain-end cleavage, during which the polymer chain breaks at a random point in the backbone, resulting in a gradual molar mass decrease [57,58]. A further drop in the value is expected to occur after radiation sterilization at 36 kGy [8]. The M_w_ and M_n_ values of the *S-Ref*, when compared to PLA-10HAp, dropped by 62% and 44%, respectively. Given that processing and sterilization of poly(lactide)-based copolymers typically result in a reduction in molar mass, a polymer with a higher initial molecular weight was selected in the study to preserve the required material properties and ensure proper functionality in the application. In fact, the molar mass of the sterilized scaffold is in the desired range for implant application: high enough to guarantee the needed mechanical performance, and sufficiently low to favor bioresorption at reasonable rates [59].

Filler amount was also measured, by determining the recovery (%) value via OMNISEC software, using the refractive index increment parameter (dn/dc), a unique value for each polymer–eluent pair. In the specific case of this work, it was assessed as 0.0252 mL g^−1^ basing on the pure copolymer of poly(l-lactide-*co*-d,l-lactide) in a ratio of 80/20 (Purasorb PLDL 8058, Corbion). With this value, it was possible to determine the exact amount of injected sample obtained after the filtration step holding back particles larger than 220 nm (HAp particles). For all cases, the mass loss is 10%, corresponding to HAp amount initially added upon processing, proving the efficiency of the melt-mixing process.

**Figure 4:**
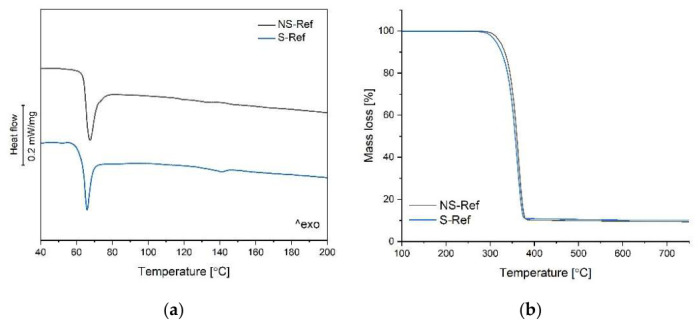
Thermal analysis of 3D-printed sample subjected to radiation sterilization, compared to non-sterilized sample: (**a**) heat flow rate upon heating; (**b**) mass loss in a function of temperature in nitrogen atmosphere.

To track the influence of the manufacturing and radiation sterilization (at 36 kGy) processes on structural properties of the 3D-printed implants, differential scanning calorimetry (DSC) data were compared for the non-sterilized and sterilized samples. The heat flow rate plots registered at the first heating scan at 10 K min^−1^ are depicted in Figure 4a, with main parameters summarized in Table 4. The DSC plot of the *NS-Ref* sample displays a glass transition (T_g_) at 65 °C coupled with an enthalpy relaxation endotherm, with no traces of cold crystallization or melting upon heating, proving that the sample is initially amorphous and remains fully amorphous thorough the analysis. The sterilization process slightly influenced the thermal behavior of the PLA composite, as depicted in the heat flow rate plot of the *S-Ref* sample: the heat flow curve reveals a glass transition at 65 °C, similar to that of the non-sterilized sample, also accompanied by an enthalpy relaxation peak, followed by a broad and weak exotherm centered around 100–110 °C due to cold crystallization and by an additional tiny endothermic peak upon further heating, centered at 141 °C, which discloses melting of the crystals grown upon heating. The thermal profile in the sterilized sample is varied due to the shortening of the polymeric chains of polylactide copolymer, quantified by GPC, which facilitates chain ordering and crystal formation upon heating [60].

The thermal analysis was completed by thermogravimetry measurements, which allowed to monitor the thermal stability of the samples. Figure 4b compares mass loss–temperature profiles of non-sterilized and sterilized 3D-printed reference samples. The *NS-Ref* sample undergoes a one-stage thermal decomposition at 341 °C, typical for polylactide-based materials [61,62]. The thermogravimetric curve recorded for the *S-Ref* sample exhibits a similar single-step degradation pattern, with a lower onset temperature (T_o_) of 337 °C compared to the non-sterilized sample (T_o_ = 341 °C). The drop in thermal stability is due to the partial decomposition of the polylactide matrix, evidenced by GPC analysis. Worth pointing out is the fact that the filler (10%wt of HAp) added to the composition is stable in the tested temperature range, whereas the polymer matrix decomposes entirely upon heating up to 600 °C [63]. It leads to the formation of sizeable residual mass (~10 wt.%) originating from the presence of a filler in the analyzed samples, as depicted in Figure 4b. This outcome is in line with the filler content identified in µCT measurements.

Physical properties of the samples were determined via density and Shore hardness tests, as presented in Table 5. The compression-molded PLA-10HAp sample was tested to assess the density of a solid material to be used as a reference in porosity and compactness calculations, as per Equations (2) and (3). As expected, a drop in density from 1.29 to 1.23 g/cm^3^ was noted for the *NS-Ref* sample, in comparison to the PLA-HAp sample, resulting from the change in internal structure of the sample caused by the manufacturing process. The process of 3D printing involves creation of an object layer-by-layer which generates voids in the structure, despite applying a 100% infill parameter during manufacturing [64]. This is proven by the calculated porosity of 4.4% with a resulting compactness of 95.4% for the *NS-Ref* sample, as presented in Table 5. Additionally, a decrease in hardness from 81 to 72°ShD was noted for the *NS-Ref* sample, as compared to the PLA-HAp sample, in accordance with the rationale discussed above. Strictly speaking, the ISO 868 norm is not recommended for porous materials, as it is meant for determination of indentation hardness of solid plastics [48]. However, due to the low porosity of the 3D-printed sample and more importantly to ensure the comparability of test results between different manufacturing processes, the Shore hardness method was used for both compression-molded and 3D-printed samples. Radiation sterilization of the sample (*S-Ref*) did not further influence the changes in density nor hardness, as expected.

Overall, the experimental data presented and discussed here confirm the suitability of the processed and sterilized implants as bone substitute allographs. Patient-tailored personalized alloplasts were produced and implanted, and the influence of prolonged contact with human body on physicochemical properties of the alloplasts is detailed below.

### 4.2. Bioresorption and Regrowth

**Figure 5:**
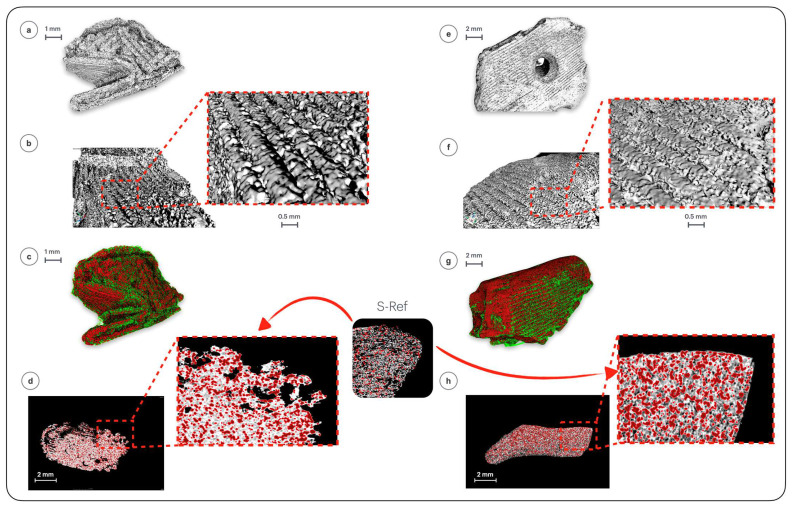
X-ray micro-computed tomography images of the *Imp-4m* and *Imp-18m*: (**a**,**e**) implants’ geometries, (**b**,**f**) implant surface, (**c**,**g**) implant geometry with visible matrix (green) and filler (red) distribution, (**d**,**h**) implant intersection together with the filler distribution (red).

**Figure 6:**
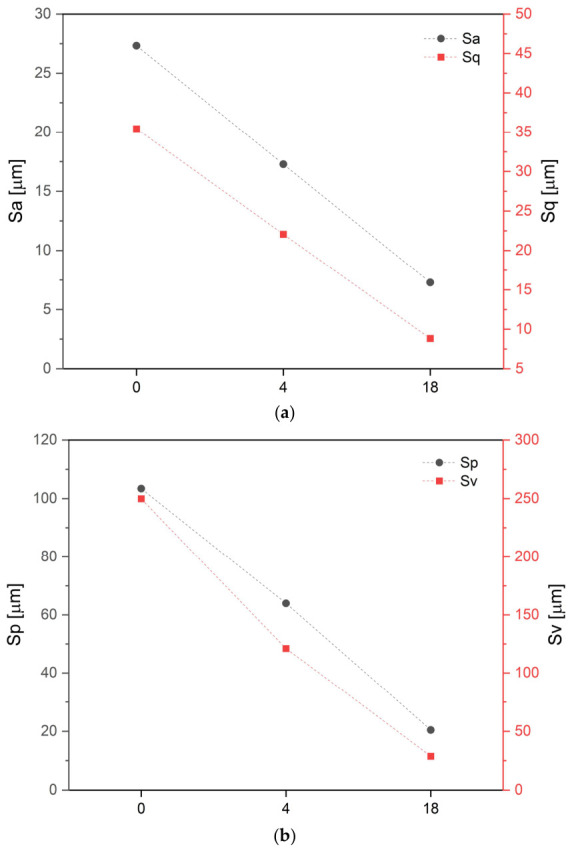

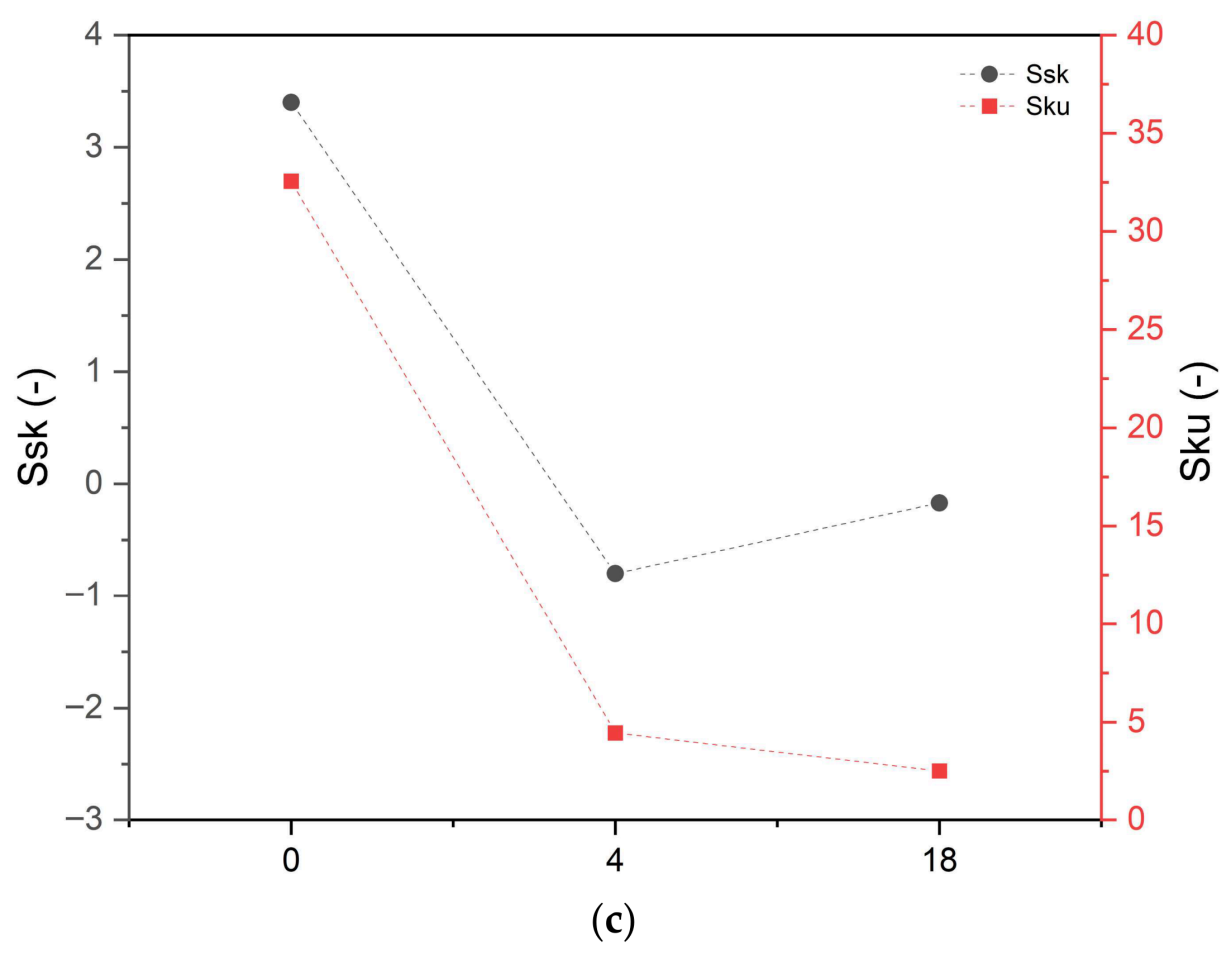
Surface topography parameters in a function of bioresorption time: (**a**) arithmetical mean height (Sa) and root mean square height (Sq) parameters; (**b**) maximum peak height (Sp) and maximum pit depth (Sv); (**c**) skewness (Ssk) in the left y-axis and kurtosis (Sku) in the right y-axis.

To track the influence of in vivo bioresorption of the implants after different degradation times, a wide range of analyses were conducted. Firstly, the implants were tested using X-ray micro-computed tomography (µCT) to assess the changes in surface topography and the interiors of the implants subjected to contact with human body for 4 and 18 months. The changes in surface morphology, exhibited in Figure 5, are quantified in Figure 6 as a variation in height parameters with implantation time.

Visual comparison of surface topography of the implants maintained in the human body for 4 (Figure 5b) and 18 months (Figure 5f) with the non-implanted material (Figure 3b) evidences a gradual smoothing of the surface. This was quantified by numerical analysis which revealed a progressive decrease in statistical and extreme surface height parameters over time compared to the reference sample, indicating a significant loss of surface peculiarities: root mean square height (Sq) decreased from 35.4 to 8.8 µm, arithmetical mean height (Sa) was reduced from 27.3 to 7.3 µm, maximum peak height (Sp) declined from 103.4 to 20.4 µm, maximum pit depth (Sv) decreased from 249.9 to 28.7 µm, kurtosis (Sku) decreased from 32.6 to 2.5, and skewness (Ssk) dropped from 3.4 for the *S-Ref* to negative values, specifically −0.80 for *Imp-4m* and −0.17 for *Imp-18m* (Figure 6). The consistent decrease in these values highlighted that after implantation the surfaces exhibited a gently undulating topography, transitioning from valley-like features with high-aspect-ratio projections towards a flatter morphology, as visible in Figure 5. The varied surface topography results from both processes of surface dissolution (biodegradation) and coverage by newly formed tissue: after implantation, microgaps and irregularities on the implant surface are filled with organic deposits or mineralized tissue, while at the same time the peak height decreases due to polylactide resorption. This represents biological transformation of the surface, a marker of bone tissue formation integrating with the implant.

**Figure 7:**
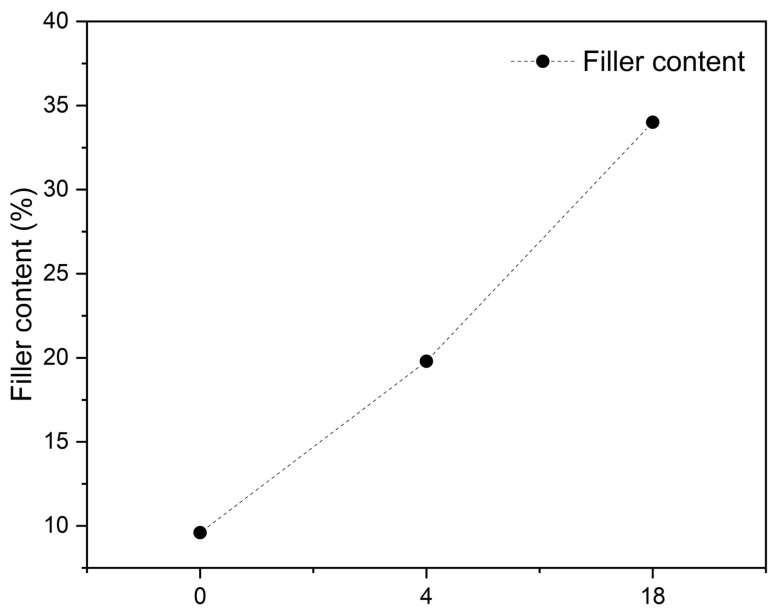
Filler content in a function of bioresorption time.

The internal structures of the implants were also analyzed to study the evolution of filler content. In the reference material, i.e., the non-implanted sample, the filler appeared as regularly and uniformly distributed spheres, with clear boundaries with the matrix. Over time, significant changes in matrix–filler morphology were observed within the samples, as shown in the bottom part of Figure 5. An increase in the filler’s proportion, quantified in Figure 7, probes its biological activation and potential growth, which results from the action of the biological environment and signals its involvement in regenerative processes. The significant increase in filler-to-matrix ratio between months 4 and 18 suggests progressive buildup of new tissues around the filler. The initially spherical and homogeneous filler particles became overgrown, losing their symmetry, reflecting potential resorption, and the buildup of new tissue around the filler. The latter is also ascertained by filler optical blurring and contour changes, as visible in Figure 5h. The recorded increase in the filler’s percentage share (from 9.6%, for the reference sample, up to 19.8 and 34%, respectively), seen in Figure 7, results from the adhesion of new tissue to filler surface indicating its bioactivity and effectiveness in stimulating regeneration.

**Figure 8:**
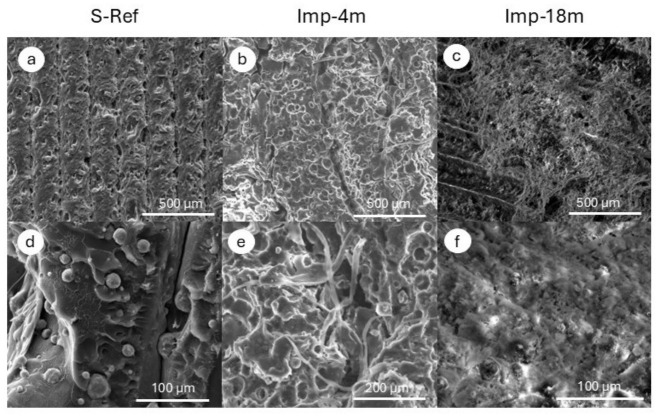
SEM micrographs of the *S-Ref* (**a**,**d**), *Imp-4m* (**b**,**e**), and *Imp-18m* (**c**,**f**) samples.

To substantiate the results of µCT measurements, additional surface and cross-sectional analysis via scanning electron microscopy (SEM) was performed for all the samples, as presented in Figure 8. The *S-Ref* sample surface (Figure 8a) reveals a strongly undulated structure, with visible extrusion paths accompanied by depressions, voids and microgaps between them. These features are typical of FDM-manufactured components, also seen in Figure 3b, and show the sequence of deposited layers [64], which results in a porous structure, as also evidenced by micro-tomography images and density measurements. The SEM micrograph of the *S-Ref* sample cross-section, as depicted in Figure 8b, indicates a globular morphology and agglomeration behavior of HAp particles. The average particle size was determined from dozens of measurements from SEM images as follows: the size of sphere-like shape particles ranged from 58.3 to 1189 nm, giving an average value of 510.4 ± 314 nm.

Major changes were noted for the implants subjected to bioresorption for 4 and 18 months. The *Imp-4m* sample exhibits the extrusion paths typical of FDM, however deformed with a substantial number of voids on the surface, appearing because of polymeric matrix dissolution (Figure 8b) [59]. Also, additional fibrous structures, not present in the reference sample, were recorded on the sample surface (Figure 8e). The *Imp-18m* sample also revealed a wavy surface morphology, however partially covered with overgrown, stable tissue, as presented in Figure 8c. The magnification of this tissue, as presented in Figure 8f, is quite smooth. The newly forming tissue gradually covered the valley features of the 3D-printed implant, leading to their progressive masking and smoothing of the surface topography. Over time, this process indicated an active remodeling of the implant. Importantly, the newly formed tissue demonstrated intimate integration with the implant surface, which may reflect favorable conditions for long-term stability and functionality of the implant. The hydroxyapatite (HAp) particles remained observable within the surface, despite the fact that they became gradually overgrown and firmly embedded in the tissue. Such incorporation of HAp into the forming structure highlights not only the osteoconductive properties of the material, but also its active participation in the integration process, potentially enhancing the biological performance of the implant [65].

**Figure 9:**
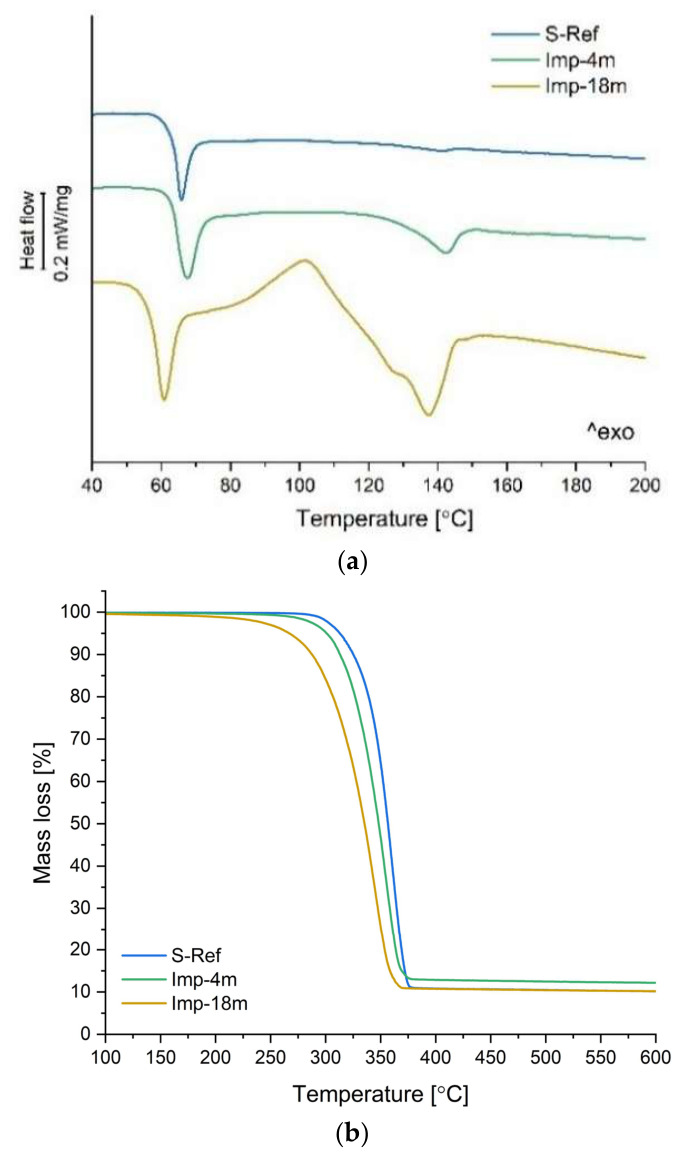

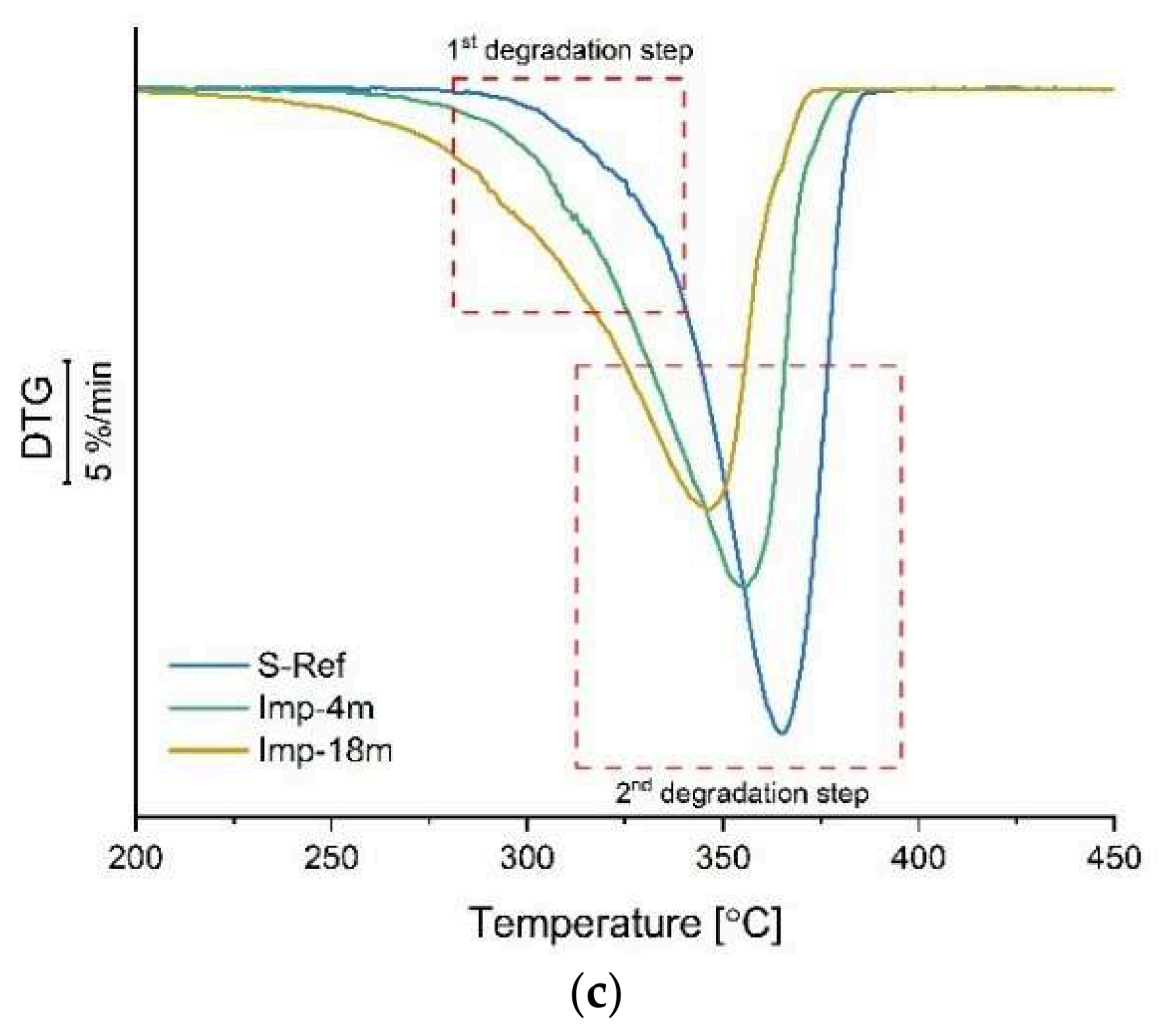
Thermal analysis of 3D-printed implants subjected to in vivo bioresorption, compared to sterilized sample: (**a**) heat flow rate upon heating; (**b**) mass loss in a function of temperature in nitrogen atmosphere; (**c**) first derivative of mass loss curves in a function of temperature.

Thermal analysis of the implant samples was conducted by differential scanning calorimetry, with the heat flow rate plots registered at the first heating scan at 10 K min^−1^, depicted in Figure 9a and summarized in Table 6. The DSC plot of the *Imp-4m* displays a glass transition (T_g_) at 65 °C and a barely visible trace of cold crystallization upon further heating. A major endothermic peak due to crystal melting appears at 143 °C, revealing a melting enthalpy much higher than that measured for the reference sample (4.6 vs. 0.47 J/g). A massive change in the DSC plot of the *Imp-18m* sample was noted, when compared to both the *S-Ref* and *Imp-4m* samples. A decrease down to 60 °C in the T_g_ value was noted for the *Imp-18m* sample. Additionally, an intense cold crystallization peak appeared at 102 °C, followed by melting peaks of α’- and α- crystalline phases, centered at 127 °C and 137 °C, overall disclosing a melting enthalpy of 21.40 J/g. Such changes are due to the largely decreased molar mass, which enhances the motion of the polymer chains towards the growing crystals, favoring crystallization, and are also aided by the presence of additional solid particles that may act as heterogeneous nucleation sites for crystal growth [66,67].

Thermal analysis was completed by thermogravimetric measurements to monitor the thermal stability of the samples. Figure 9b,c present mass loss–temperature profiles of the implant samples subjected to in vivo bioresorption, and their first derivatives, in comparison with the sterilized 3D-printed reference sample. The *S-Ref* sample, as reported above, undergoes a one-stage thermal decomposition starting at 341 °C. The thermogravimetric curves recorded for the *Imp-4m* and *Imp-18* samples differ greatly from the reference sample, as they exhibit a double-step degradation pattern. Since the first degradation step partially overlaps with the second one, to better resolve the overlapping processes, the first derivatives of the mass loss curves were determined and are presented in Figure 9c. The initial degradation step is observed within the temperature range of 290–300 °C for the *Imp-4m* sample and 300–315 °C for the *Imp-18m* sample, whereas it is absent in the reference sample. These results correlate with the residual mass analysis, which indicates a ~2% higher residual mass for the *Imp-4m* sample compared to the *S-Ref* and *Imp-18m* samples. This increase at early implantation stages is attributed to the transient formation of metastable Ca–P phases, which are later eliminated or incorporated into a thermally stable apatite-like phase, converging to the intrinsic HAp fraction over time [68,69,70,71,72]. The onset temperature (T_o_) of the main degradation step, referring to the polylactide matrix degradation, was lowered for both implanted samples, from 337 °C down to 323 °C and 306 °C, respectively, when compared to the *S-Ref*. This drop in thermal stability is to be correlated to partial decomposition of the polylactide matrix upon contact with the human body.

Sample degradation was also quantified by measuring the molecular weights (M_w_ and M_n_) of bioresorbed samples in comparison with the reference sample (*S-Ref*) *via* gel permeation chromatography (GPC), with the results presented in Table 7. A major reduction in molar mass (M_w_) by 77%, in contrast to the PLA-10HAp sample, was observed for the *Imp-4m*. A further drop in Mw appeared for the *Imp-18m* sample, with a decrease of 92%. This drop is caused by the ongoing resorption of polylactide matrix upon contact with human body fluids for 4 and 18 months, respectively. Moreover, the recovery (%) values for both implanted samples (*Imp-4m* and *Imp-18m*) decreased, due to lower sample mass recuperated after dissolution and filtration processes. This is ascribed to exposure to bodily fluids, which resulted in Ca–P layer growth around the HAp particles throughout the implant structure, as also evidenced *via* µCT analysis.

Analysis of physical properties allowed to assess the density of the implants in a function of bioresorption time interval. Prolonged exposure of implants to the human body resulted in a gradual reduction in density from 1.24 g/cm^3^, reported for the *S-Ref*, down to 1.18 and 1.14 g/cm^3^, for the *Imp-4m* and *Imp-18m* samples, respectively. This indicates continuous regrowth of the implant structure—HAp particles are overgrowing with new tissues while polylactide vanishes at the same time—which induced changes in the density of the material. As seen in Figure 5h, the previously unoccupied spaces inside the implant structure were filled with new tissue. This demonstrates that the newly formed tissue exhibits a lower density than the PLA-based composite (<1.24 g/cm^3^), which contributes to a reduction in the overall density of the implanted samples.

Assessment of other physical properties of the *Imp-4m* and *Imp-18m* samples was not feasible due to the limited size of the implant fragments available for analysis. However, during the second surgical procedure involving *Imp-18m*, i.e., the insertion of titanium dental implants for full dental rehabilitation, the polymeric implant fractured while drilling holes in the PLA-based implant using a trephine bur. Notably, similar procedures—such as drilling and mechanical manipulation—had been performed at the time of the initial implantation without any complications. The material could be processed with standard dental instruments, and these procedures did not compromise the integrity of the implant. In contrast, repeating these procedures on the implant after 18 months in vivo resulted in structural failure, indicating a substantial increase in brittleness and a loss of resistance to dynamic mechanical loads. The degradation of biodegradable polymer matrices reinforced with rigid fillers is typically accompanied by a deterioration in mechanical properties, which is primarily attributed to interfacial weakening and reduced stress transfer efficiency at the matrix–filler boundary [73,74,75,76]. Concurrently, hydrolytic degradation and mass loss processes, as supported by the experimental data presented above, promote the formation of microvoids and an overall increase in matrix porosity, which further accelerates mechanical performance decay.

## 5. Conclusions

This study investigates the effects of manufacturing and sterilization processes, followed by in vivo resorption, on the properties of customized implants fabricated from a composite material consisting of a polylactide copolymer and hydroxyapatite particles (10 wt.%). The material was designed to undergo gradual degradation in the human body and be progressively replaced by natural tissue, supporting bone defect reconstruction.

Investigations of implants retrieved after 4 and 18 months suggest the progression of resorption and tissue remodeling processes. µCT analysis indicates surface alterations, including smoothing and an apparent increase in filler content, likely associated with polymer resorption and concurrent tissue deposition. Newly formed tissue was observed both on the surface of filler particles and within the pore space of the implant, reaching approximately 34% of the implant volume after 18 months. SEM observations further support the presence of tissue formation in the vicinity of hydroxyapatite particles. Structural characterization indicates a gradual decrease in the molar mass of the polylactide copolymer, consistent with ongoing degradation.

The material also exhibited notable changes in mechanical behavior over time. While standard dental tools were successfully employed to create holes at the time of implantation, after 18 months of in vivo incubation, the implant material showed visible changes—such as discoloration and altered surface morphology. Critically, when re-drilling was attempted using a trephine for titanium implant placement, the polymer implant fractured, indicating a pronounced increase in material brittleness.

In this clinical material, the results indicate biocompatibility and the absence of adverse local reactions, as well as the material’s ability to undergo resorption and support tissue regrowth. However, due to the small sample size and being limited to two patients only, these findings should be considered preliminary and require validation in larger clinical studies.

## Figures and Tables

**Table 1:** Symbols and description of the samples.

	Sample Name	Description
Reference samples	PLA-10HAp	PLA combined with 10 wt.% HAp particles (T.2)
	*NS-Ref*	Non-sterilized 3D-printed implant (T.4)
	*S-Ref*	Sterilized 3D-printed implant (T.5)
Implants subjected to bioresorption process	*Imp-4m*	Sterilized 3D-printed implant residing in the human body for 4 months (T.6)
	*Imp-18m*	Sterilized 3D-printed implant residing in the human body for 18 months (T.6)

**Table 2:** Surface topography parameters.

Sample	Sq µm	Ssk -	Sku -	Sp µm	Sv µm	Sz µm	Sa µm	Smr %
*S-Ref*	35.43	3.4	32.56	103.40	249.90	353.40	27.33	0.0011
	Smc µm	Sdc µm	Sal Mm	Sdq -	Sdr %	Shrn -	Sdrn -	Filler content %
	36.85	83.18	0.047	1.98	39.77	0.43	0.39	9.6

**Table 3:** Weight-average molar mass (M_w_), number-average molar mass (M_n_) and recovery of the melt-mixed PLA-10HAp, as well as non-sterilized and sterilized 3D-printed samples.

Sample	M_w_ (g/mol)	M_n_ (g/mol)	Recovery %	Decrease in Mw * (%)	Decrease in Mn * (%)
PLA-10HAp	226,155	122,130	90	-	-
*NS-Ref*	163,770	104,450	90	28	14
*S-Ref*	86,540	67,920	90	62	44

* with regard to PLA-10HAp sample.

**Table 4:** Glass transition temperature (T_g_), melting temperature (T_m_), melting enthalpy (ΔH_Tm_), onset of degradation temperature (T_d’onset_), and residual mass of non-sterilized and sterilized reference samples.

Sample	T_g_ (°C)	T_m_ (°C)	ΔH_Tm_ (J/g)	T_d’onset_ (°C)	m_R_ (%)
*NS-Ref*	65	–	–	341	9.60
*S-Ref*	65	141	0.465	337	10.15

**Table 5:** Density, porosity, compactness and Shore hardness of the compression-molded PLA-10HAp, as well as non-sterilized and sterilized 3D-printed samples.

Sample	Density g/cm^3^	Porosity %	Compactness %	Shore Hardness °ShD
PLA-10Hap *	1.29 ± 0.01	-	-	81 ± 3
*NS-Ref*	1.23 ± 0.01	4.4 ± 0.6	95.4 ± 0.9	72 ± 3
*S-Ref*	1.24 ± 0.01	4.6 ± 0.9	95.6 ± 0.6	75 ± 4

* Reference sample of the PLA-based composite with 10 wt.% HAp particles, shaped via the compression molding technique, to evaluate the real density parameter.

**Table 6:** Glass transition temperature (T_g_), cold crystallization temperature (T_cc_), cold crystallization enthalpy (ΔH_Tcc_), α’-phase melting temperature (T_m1_), α-phase melting temperature (T_m2_), melting enthalpy (ΔH_Tm_), onset of degradation temperature (T_d’onset_), mass loss (m_R_) and peak maximum of the first derivative of mass loss plot in a function of temperature (DTG).

Sample	T_g_ (°C)	T_cc_ (°C)	ΔH_Tcc_ (J/g)	T_m1_ (°C)	T_m2_ (°C)	ΔH_Tm_ (J/g)	T_d’onset_ (°C)	m_R_ (%)	DTG (°C)
*S-Ref*	65	–	–	–	141	0.465	337	10.15	365
*Imp-4m*	65	–	–	–	143	4.614	323	12.18	356
*Imp-18m*	60	102	17.94	127	137	21.40	306	10.16	346

**Table 7:** Molecular weight characteristics, recovery and density data.

Sample	M_w_ (g/mol)	M_n_ (g/mol)	Recovery %	Decrease in Mw * (%)	Decrease in Mn * (%)	Density g/cm^3^
*S-Ref*	86,540	67,920	90	62	44	1.24 ± 0.01
*Imp-4m*	52,910	31,220	83	77	74	1.18 ± 0.01
*Imp-18m*	17,815	13,588	85	92	89	1.14 ± 0.06

* with regards to PLA-10HAp sample.

## Data Availability

The original contributions presented in this study are included in the article. Further inquiries can be directed to the corresponding author.
